# Low prognostic nutritional index predicts poor outcome in newly diagnosed angioimmunoblastic T-cell lymphoma

**DOI:** 10.3389/fnut.2025.1622691

**Published:** 2025-06-26

**Authors:** Renqin Li, Wei Zhang, Le Yu, Ping Wu, He Huang, Hongqiang Guo, Tongyu Lin, Huangming Hong, Huawei Weng

**Affiliations:** ^1^Department of Medical Oncology, Sichuan Cancer Hospital and Institute, Sichuan Cancer Center, Affiliated Cancer Hospital of University of Electronic Science and Technology of China, Chengdu, China; ^2^Department of Medical Oncology, State Key Laboratory of Oncology in South China, Guangdong Provincial Clinical Research Center for Cancer, Sun Yat-sen University Cancer Center, Guangzhou, China; ^3^The Affiliated Cancer Hospital of Zhengzhou University, Henan Cancer Hospital, Zhengzhou, China

**Keywords:** angioimmunoblastic T-cell lymphoma, prognostic nutritional index, lymphocyte, albumin, prognosis, risk models

## Abstract

**Background:**

Angioimmunoblastic T-cell lymphoma (AITL) is a rare subtype of peripheral T-cell lymphoma, characterized by an aggressive disease course and poor prognosis. The prognostic nutritional index (PNI), which reflects nutritional and immune status, has emerged as a potential prognostic factor in various cancers.

**Methods:**

In this multicenter retrospective study, a total of 173 patients with AITL between January 2010 and December 2022 were enrolled from three institutes in China. The optimal cutoff value for PNI was determined using the maximally selected rank statistics (MaxStat) analysis. The association of PNI and overall survival (OS) or progression free survival (PFS) was evaluated in univariable and multivariable Cox regression analyses. Receiver operating characteristic (ROC) curves were used to evaluate the prognostic performance and predictive accuracy of PNI combined with International Prognostic Index (IPI) and Prognostic Index for T-cell lymphoma (PIT).

**Results:**

Based on the MaxStat analysis, a score of 40.8 was identified as the optimal cutoff value for the PNI. Survival analysis revealed that the low PNI group had worse OS and PFS. The 3-year OS and PFS for the low PNI group were 27.5 and 26.5%, respectively, compared to 84.7 and 74.4% for the high PNI group (*p* < 0.001). Multivariate analyses indicated that PNI was significantly associated with both OS (HR 0.221, 95% CI 0.128–0.381, *p* < 0.001) and PFS (HR 0.380, 95% CI 0.242–0.596; *p* < 0.001). We further integrated PNI into the IPI and PIT prognostic models, and the predictive accuracy of both models was significantly improved.

**Conclusion:**

PNI is a simple and easily accessible prognostic indicator for AITL.

## Introduction

Angioimmunoblastic T-cell lymphoma (AITL) is a rare subtype of peripheral T-cell lymphoma (PTCL), representing approximately 1–2% of non-Hodgkin lymphoma cases and 15–20% of PTCLs (1–3). AITL generally displays an aggressive disease course and poor prognosis with standard anthracycline-based chemotherapy regimens (4). Currently, the International Prognostic Index (IPI) (5) and the Prognostic Index for T-cell lymphoma (PIT) (6) are commonly used prognostic models for AITL. However, these models have shown limited accuracy in predicting outcomes in AITL patients (4, 7). It is essential to identify additional prognostic markers to enhance risk stratification and guide treatment decisions in AITL.

Numerous studies have demonstrated a close association between the nutritional and immune status and cancer prognosis (8–10). In particular, patients with AITL are often characterized by malnutrition and immune dysregulation due to the biological characteristics of the disease (11). The prognostic nutritional index (PNI) is derived from serum albumin and lymphocyte counts in peripheral blood. It reflects the nutritional and immunological status. This indicator was initially introduced to predict operative risk in gastrointestinal surgery (12). In recent years, several studies have demonstrated that low PNI has been associated with dismal outcomes in various types of cancer, including lymphoma (8, 13, 14). However, the association between PNI and the prognosis of AITL patients has not yet been elucidated.

Herein, we conducted a multicenter retrospective study to evaluate the prognostic value of PNI in patients with AITL.

## Materials and methods

### Patients

In this study, we retrospectively reviewed the records of all newly diagnosed AITL patients across three institutes in China, from January 2010 to December 2022, with diagnoses confirmed according to the World Health Organization classification of hematopoietic and lymphoid tumors. Collected data included baseline clinical characteristics, laboratory results, and treatment regimens. All patients were staged according to the Ann Arbor staging system. Initial treatment was administered at the discretion of local clinicians. The PNI was calculated as serum albumin (g/L) + 5 *Χ* absolute lymphocyte count (10^9^/L). This study was approved by the institutional reviewed board and was conducted in accordance with the Declaration of Helsinki.

### Statistical analysis

Pearson’s χ^2^ tests or Fisher’s exact tests were used to compare the categorical variables. The Mann–Whitney tests were used to compare the continuous variables. Overall survival (OS) was defined as the time from initial diagnosis to death from any cause or to the last follow-up. Progression-free survival (PFS) was defined as the time from initial diagnosis to disease progression, relapse, or death from any cause. The optimal cutoff point for PNI was determined using the maximally selected rank statistics (MaxStat) analysis. Survival estimates were calculated using Kaplan–Meier method, and survival curves were compared with log–rank tests. Multivariate analysis was conducted using Cox regression, including variables with a *p* value ≤ 0.1 in univariate analysis. To evaluate the predictive accuracy of prognostic models, receiver operating characteristic (ROC) curves were generated, and the corresponding areas under the curves (AUCs) were calculated. DeLong’s test was used to compare the predictive performance of the models. A two-tailed *p* value of < 0.05 was considered statistically significant. All statistical analyses were conducted using SPSS statistics version 26.0 (IBM Corporation) and R software version 4.4.1 (R Foundation).

## Results

### Clinical characteristics of AITL patients

A total of 173 newly diagnosed patients with AITL were included and analyzed in this study. The baseline clinical characteristics of the patients are detailed in Table 1. The median age at diagnosis was 62 years (range, 20–84 years), with 55.5% of patients being older than 60 years. In terms of disease stage, 157 patients (90.8%) presented with stage III or stage IV disease. B symptoms (defined as recurrent fever, night sweats, or >10% weight loss) were presented in 98 patients (56.6%). Based on the IPI and the PIT models, 54.4% (IPI ≥ 3) and 57.2% (PIT ≥ 2) of patients were stratified into the high-risk group.

**Table 1:** Patient characteristics.

Characteristics	*N* = 173	(%)
Age median	62	
≥60	96	55.5
Male	115	66.5
Stage III–IV	157	90.8
ECOG > 2	47	27.2
B symptoms	98	56.6
Extranodal sites > 1	45	26.0
Bone marrow involvement	45	26.0
LDH > ULN	113	65.3
Platelet ≤ 150 × 10^9^/L	38	22.0
White cell ≤ 5.0 × 10^9^/L	46	26.6
Hemoglobin ≤ 120 g/L	85	49.1
Albumin < 35 g/L	61	35.3
IPI score		
0/1	25	14.5
2	54	31.2
3	54	31.2
4/5	40	23.2
PIT score		
0	14	8.1
1	60	34.7
2	58	33.5
3/4	41	23.7

ECOG, Eastern Cooperative Oncology Group; LDH, lactate dehydrogenase; ULN, upper limit of normal value; IPI, International Prognostic Index; PIT, prognostic index for T-cell lymphoma.

### Treatment and outcome

The majority of patients (86.7%) received anthracycline-based chemotherapy. Among them, 85 patients (49.1%) were treated with the CHOP regimen (cyclophosphamide, doxorubicin, vincristine, and prednisolone), and 65 patients (37.6%) were treated with the CHOEP regimen (cyclophosphamide, doxorubicin, vincristine, etoposide, and prednisolone). The remaining patients underwent either non-anthracycline chemotherapy regimens (*n* = 18, 10.4%) or received supportive care alone (*n* = 5, 2.9%). Chidamide, a histone deacetylase inhibitor (HDACi), was administered in combination with chemotherapy for 26 patients (15.0%). Additionally, autologous stem cell transplantation (ASCT) was performed in 8 patients (4.6%) as consolidation therapy following their first remission.

**Figure 1:**
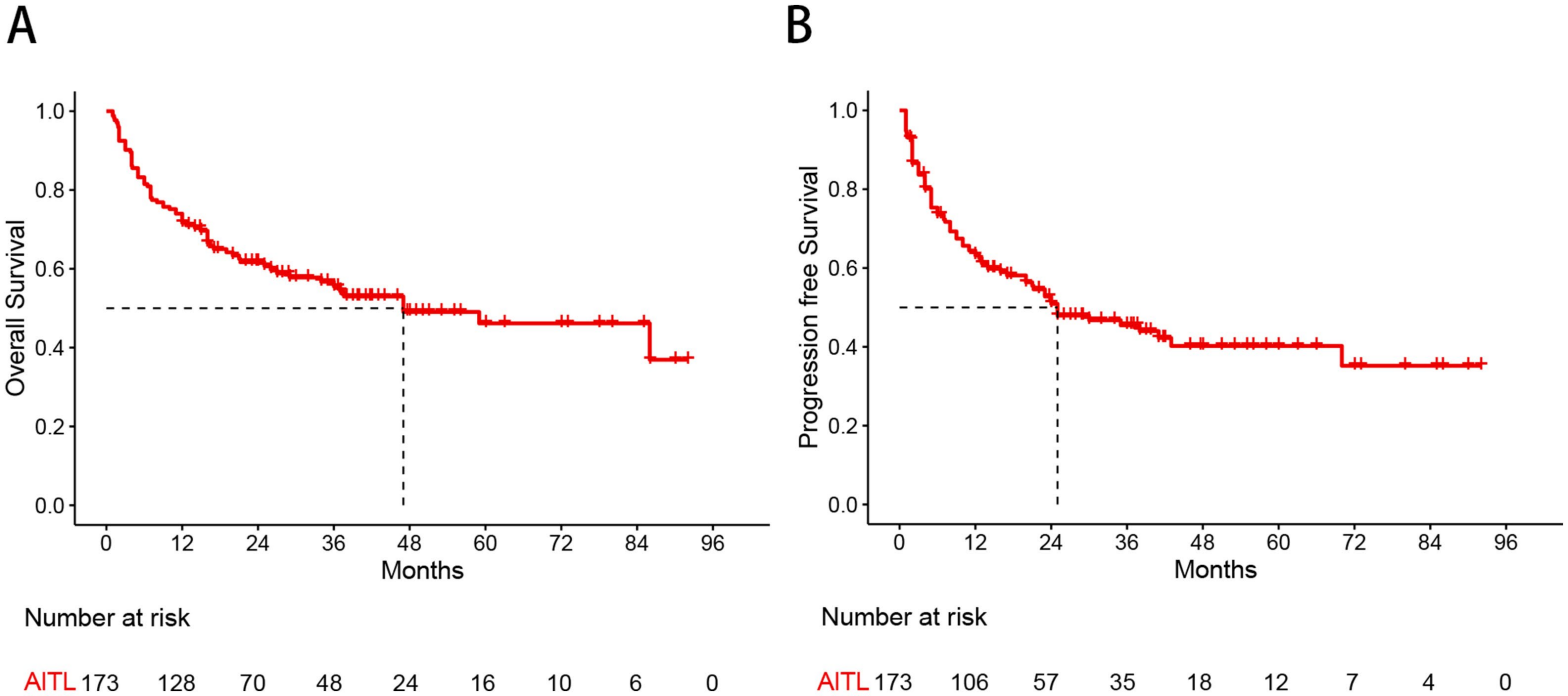
Kaplan–Meier curves show overall survival **(A)** and progression free survival **(B)** estimates for the entire study cohort.

With a median follow-up of 37.8 months, the 3-year and 5-year OS rates for the whole group were 55.5 and 46.2%, respectively, while the 3-year and 5-year PFS rates were 45.5 and 40.0%, respectively (Figure 1). There were no significant differences in OS (*p* = 0.924) or PFS (*p* = 0.770) between patients treated with the CHOP and CHOEP regimens.

### Prognostic nutritional index

**Figure 2:**
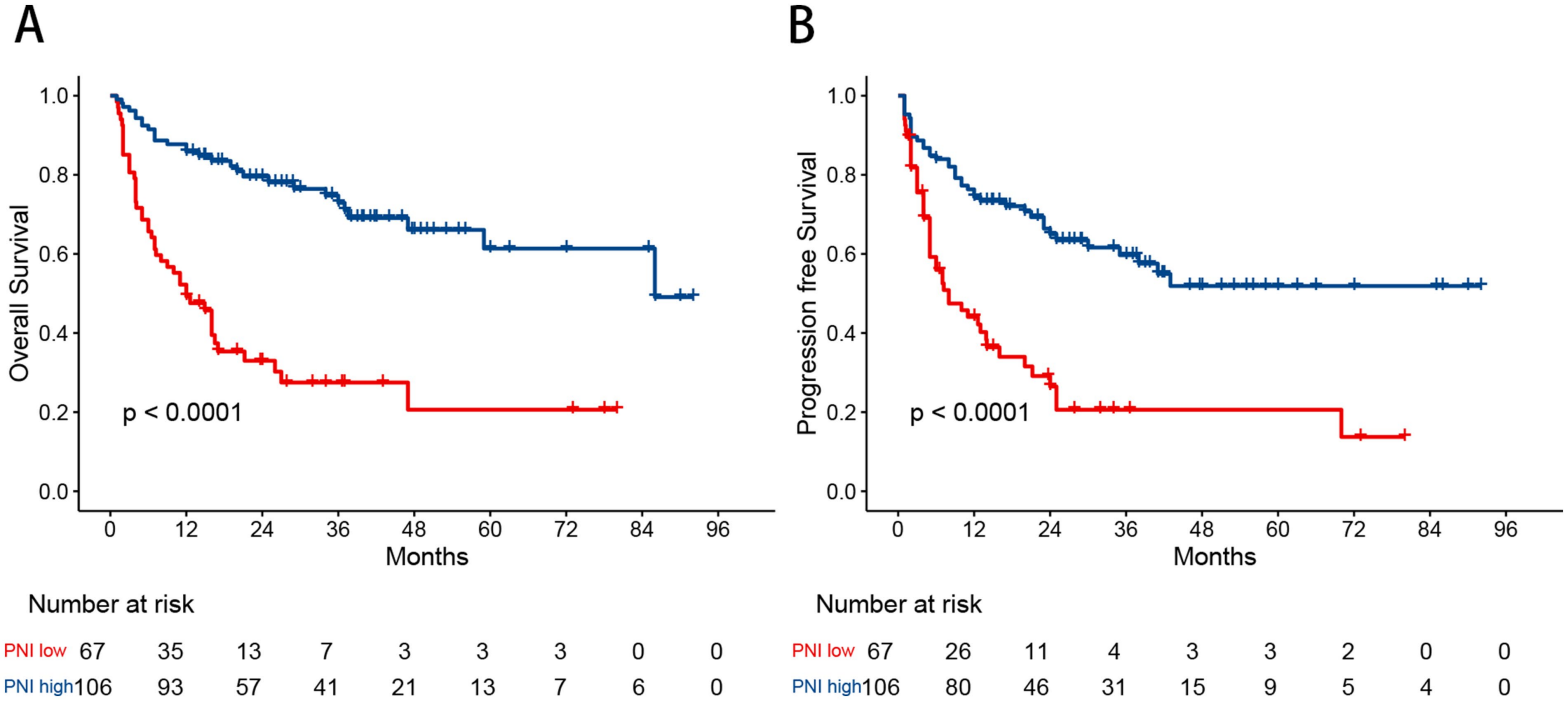
Kaplan–Meier curves for overall survival **(A)** and progression free survival **(B)** in patients with angioimmunoblastic T-cell lymphoma stratified by the prognostic nutritional index.

The optimal cutoff value for the PNI was determined using MaxStat analysis, which identified a threshold of 40.8 as the most effective value for distinguishing between two prognostic groups (Supplementary Figure S1). Based on this cutoff value, 67 patients (38.7%) were classified into the low PNI group, and 106 patients (61.3%) into the high PNI group. Low PNI was found to be significantly associated with Ann Arbor stage III/IV, poor performance status (PS), the presence of B symptoms, elevated lactate dehydrogenase (LDH) levels, bone marrow involvement, hemoglobin concentration, and albumin levels. A comparison of baseline characteristics between the PNI groups is shown in Table 2. In the Kaplan–Meier analysis, the low PNI group had significantly worse 3-year OS (27.5% vs. 84.7%, *p* < 0.001) and PFS (26.5% vs. 74.4%, *p* < 0.001) compared to the high PNI group (Figure 2).

**Table 2:** Comparison of patient characteristics between the prognostic nutritional index stratifications.

Characteristics	PNI low (≤40.8)	PNI high (>40.8)	*p* value
No. of patients	67	106	
Age ≥ 60	42 (62.7)	54 (50.9)	0.130
Male	44 (65.7)	71 (67.0)	0.859
Stage III–IV	65 (97.0)	92 (86.8)	0.024
ECOG > 2	24 (35.8)	23 (21.7)	0.042
B symptoms	47 (70.1)	51 (48.1)	0.004
Extranodal sites > 1	20 (29.9)	25 (23.6)	0.360
Bone marrow involvement	12 (17.9)	34 (32.1)	0.040
LDH > ULN	55 (82.1)	61 (57.5)	0.001
Platelet ≤ 150 × 10^9^/L	19 (28.4)	19 (17.9)	0.106
White cell ≤ 5.0 × 10^9^/L	23 (34.3)	23 (21.7)	0.067
Hemoglobin ≤ 120 g/L	47 (70.1)	43 (40.6)	<0.001
Albumin < 35 g/L	45 (67.2)	16 (15.1)	<0.001
IPI score			0.006
0/1	2 (3.0)	23 (21.7)	
2	21 (31.3)	33 (31.1)	
3	21 (31.3)	33 (31.1)	
4/5	23 (34.3)	17 (16.1)	
PIT score			0.019
0	0	14 (13.2)	
1	23 (34.3)	37 (34.9)	
2	26 (38.8)	32 (30.2)	
3/4	18 (26.9)	23 (21.7)	

ECOG, Eastern Cooperative Oncology Group; LDH, lactate dehydrogenase; ULN, Upper limit of normal, IPI, International Prognostic Index; PIT, prognostic index for T-cell lymphoma; PNI, prognostic nutritional index.

### Clinical prognostic factors

Analyses of prognostic factors are summarized in Table 3 and Supplementary Figure S2. In univariate analyses, age, Eastern Cooperative Oncology Group (ECOG) performance status, bone marrow involvement, elevated LDH levels, platelet counts, white cell counts, and low PNI were identified as adverse prognostic factors for OS. ECOG performance status, elevated LDH levels, platelet counts, white cell counts and low PNI were adverse predictors for PFS.

**Table 3:** Univariate and multivariate Cox regression analyses of OS and PFS.

Outcomes	Characteristics	Univariate		Multivariate	
		HR (95% CI)	*p* value	HR (95% CI)	*p* value
OS	Age	1.649 (1.017–2.673)	0.043		
	Sex	0.768 (0.463–1.275)	0.307		
	Stage III–IV	2.073 (0.756–5.683)	0.156		
	ECOG > 2	2.961 (1.843–4.756)	<0.001	2.668 (1.608–4.426)	<0.001
	B symptoms	1.483 (0.925–2.380)	0.102		
	Extranodal sites > 1	1.435 (0.874–2.356)	0.153		
	Bone marrow involvement	1.706 (1.051–2.769)	0.031	2.814 (1.662–4.767)	<0.001
	LDH > ULN	2.621 (1.474–4.659)	0.001		
	Platelet ≤ 150 ×10^9^/L	2.369 (1.437–3.906)	0.001		
	White cell ≤ 5.0 × 10^9^/L	2.199 (1.377–3.512)	0.001		
	Hemoglobin ≤ 120 g/L	1.560 (0.980–2.482)	0.061		
	PNI	0.240 (0.148–0.388)	<0.001	0.221 (0.128–0.381)	<0.001
PFS	Age	1.386 (0.897–2.142)	0.142		
	Sex	0.885 (0.560–1.397)	0.599		
	Stage III–IV	2.408 (0.882–6.572)	0.068		
	ECOG > 2	2.511 (1.602–3.937)	<0.001	2.209 (1.372–3.556)	0.001
	B symptoms	1.335 (0.865–2.060)	0.189		
	Extranodal sites > 1	1.362 (0.854–2.173)	0.195		
	Bone marrow involvement	1.337 (0.838–2.134)	0.223		
	LDH > ULN	2.079 (1.265–3.415)	0.004		
	Platelet ≤ 150 × 10^9^/L	2.431 (1.521–3.877)	<0.001	1.983 (1.212–3.245)	0.006
	White cell ≤ 5.0 × 10^9^/L	2.113 (1.364–3.276)	0.001	1.814 (1.153–2.854)	0.010
	Hemoglobin ≤ 120 g/L	1.354 (0.883–2.075)	0.165		
	PNI	0.340 (0.221–0.524)	<0.001	0.380 (0.242–0.596)	<0.001

OS, overall survival; PFS, progression free survival; HR, hazard ratio; CI, confidence interval; ECOG, Eastern Cooperative Oncology Group; LDH, lactate dehydrogenase; ULN, upper limit of normal value; PNI, prognostic nutritional index.

In multivariate analyses, ECOG performance status (hazard ratio [HR] = 2.668, 95% confidence interval [CI] 1.608–4.426; *p* < 0.001), bone marrow involvement (HR = 2.814, 95% CI 1.662–4.767; *p* < 0.001) and low PNI (HR = 0.221, 95% CI 0.128–0.381, *p* < 0.001) were significant prognostic factors for OS. For PFS, significant prognostic factors included ECOG PS (HR = 2.209, 95%CI 1.372–3.556; *p* = 0.001), platelet counts (HR = 1.983, 95%CI 1.212–3.245; *p* = 0.006), white cell counts (HR = 1.814, 95% CI 1.153–2.854; *p* = 0.010) and low PNI (HR = 0.380, 95% CI 0.242–0.596; *p* < 0.001).

### A better prognostic model based on the PIT

**Figure 3:**
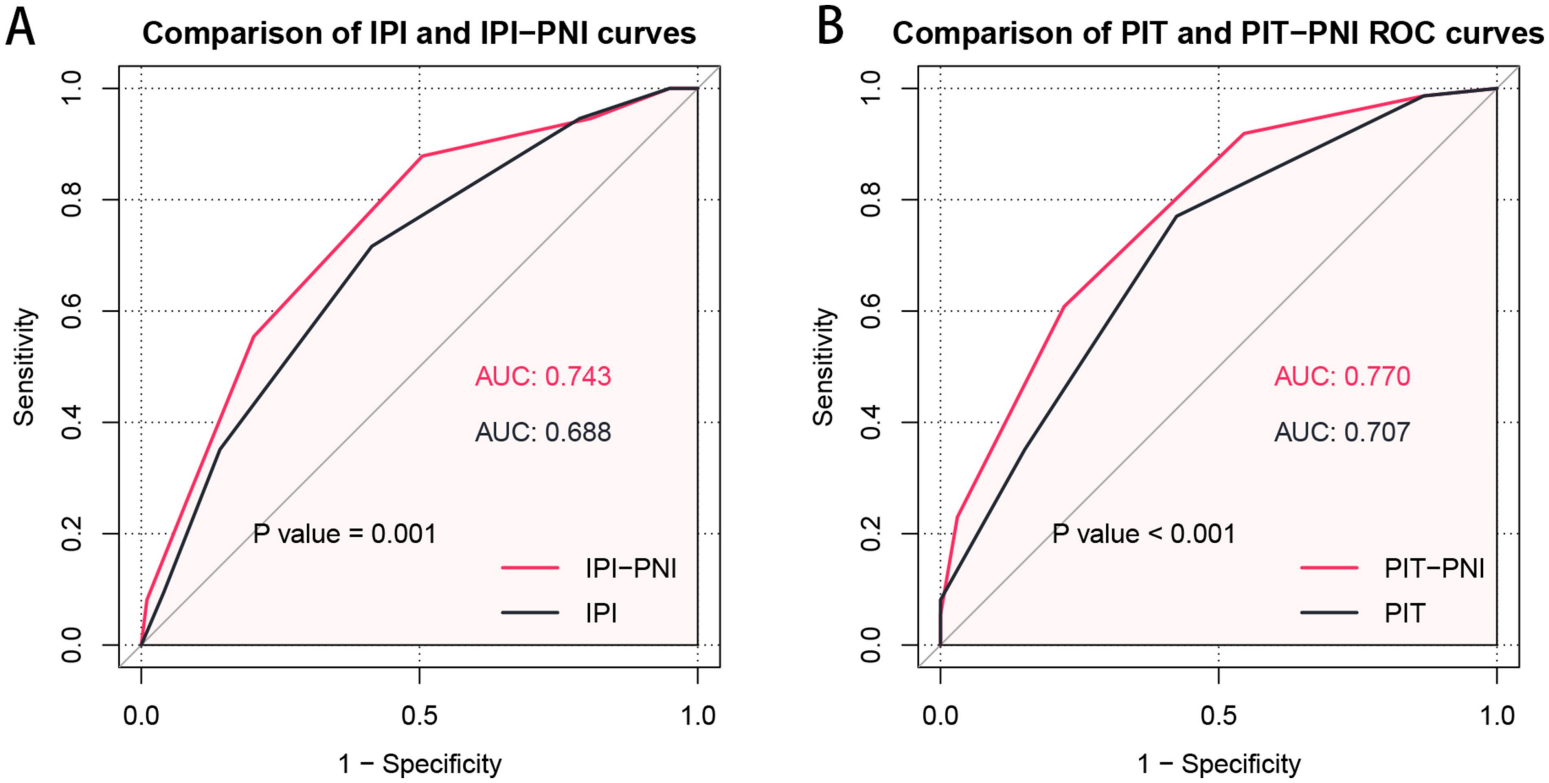
Comparison of prognostic significance of the IPI with IPI-PNI **(A)** and the PIT with PIT-PNI **(B)** by ROC curves.

The ROC curves were utilized to assess the discriminatory performance of various prognostic models by comparing their AUCs. For OS, the AUC was 0.688 (95% CI: 0.612–0.764) for the IPI model and 0.707 (95% CI: 0.633–0.780) for the PIT model. To further investigate the prognostic value of the PNI in AITL patients, it was incorporated into both the IPI and PIT models. The resulting IPI-PNI model achieved an AUC of 0.743 (95% CI: 0.671–0.815) for predicting OS, while the PIT-PNI model achieved an AUC of 0.770 (95% CI: 0.703–0.836). Both the IPI-PNI and PIT-PNI models demonstrated significantly enhanced prognostic performance compared to the IPI (*p* = 0.001) and PIT (*p* < 0.001) models alone (Figure 3).

## Discussion

In this multicenter study, we retrospectively analyzed the clinical factors and prognosis of 173 newly diagnosed AITL patients. To the best of our knowledge, this is the first study to assess the prognostic significance of PNI in newly diagnosed AITL. Our findings demonstrated that a low PNI was linked to an inferior prognosis, suggesting that PNI could serve as a valuable marker for identifying patients at higher risk of adverse clinical outcomes. Integrating PNI into existing prognostic models may enhance risk stratification and improve individualized patient management.

AITL exemplifies a neoplasm characterized by intense inflammatory and immune reactions. Consistent with previous reports (3, 4, 15), we found that AITL predominantly affects older adults (median age: 62 years) and is commonly diagnosed at an advanced stage (90.8%), often accompanied by B symptoms (56.6%), elevated LDH (65.3%), and bone marrow involvement (26.0%). Although AITL exhibits the most variable clinical course among PTCL subtypes, the overall prognosis remains poor, with 5-year OS and PFS rates ranging from 32–41% and 18–38%, respectively (3, 4, 7, 11, 15, 16). In our study, the 5-year OS and PFS rates were 46.2 and 40.0%, slightly better than previous reports. This difference may be due to some patients receiving treatment with new targeted drugs, such as Chidamide. These findings underscore the urgent need for simple, accessible, and cost-effective prognostic biomarkers to improve risk stratification and patient outcomes.

Nutritional status and inflammation have been indicated to have a strong impact on treatment tolerance and cancer progression (17). Recent studies have shown that the PNI is correlated with survival outcomes in different subtypes of lymphoma (18–21). However, its prognostic value in AITL has not been previously assessed. In our study, baseline PNI was confirmed to be an independent prognostic marker for AITL. Compared to high PNI, low PNI was significantly associated with both inferior OS and PFS in multivariate Cox regression analysis. Furthermore, integrating PNI with the IPI and PIT enhanced OS prediction accuracy. Given the clinical advantages of albumin and lymphocyte count assessments—such as reproducibility, standardization, and cost-effectiveness, PNI holds substantial potential for improving prognostic prediction in AITL.

Considering the poor outcomes of AITL with standard CHOP-based therapy—particularly among high-risk patients—alternative chemotherapy backbones or the incorporation of novel agents such as brentuximab vedotin, histone deacetylase inhibitors, or JAK/STAT pathway inhibitors into frontline treatment may offer clinical benefit (22–24). Our findings suggest that integrated models such as PNI-IPI and PNI-PIT can more accurately identify high-risk patients, thereby facilitating more personalized and risk-adapted therapeutic strategies in clinical practice. These hypotheses need to be confirmed by future prospective studies.

The optimal cutoff value for the PNI is usually estimated using ROC curves, which previous studies have reported to be between 40 and 50 (14, 20, 21). However, traditional ROC analysis focuses solely on survival outcomes without accounting for survival time, potentially introducing bias. In this study, we utilized MaxStat analysis, which identified 40.8 as the optimal cutoff value, providing a more precise stratification of patients. Patients with a low PNI were more likely to exhibit Ann Arbor stage III/IV, poor performance status, B symptoms, bone marrow involvement, and elevated LDH. These results suggest that PNI reflects tumor burden and may serve as a useful prognostic marker in clinical practice.

Our study has several limitations. First, due to its retrospective nature, selection bias was unavoidable. Additionally, the rarity of AITL resulted in a limited sample size, and an independent validation cohort was not utilized to verify our findings. Although a strong association between low PNI and poor prognosis was observed, the underlying mechanisms are still uncertain.

In conclusion, our study demonstrated that the PNI is an independent prognostic factor for outcomes of AITL. Incorporating the PNI into the current prognostic models could improve risk stratification, facilitating more tailored and effective treatment strategies. Further research is essential to explore the clinical relevance and underlying biological pathways through which PNI impacts disease progression in AITL.

## Data Availability

The raw data supporting the conclusions of this article will be made available by the authors, without undue reservation.

## References

[1] Lunning MA Vose JM. Angioimmunoblastic T-cell lymphoma: the many-faced lymphoma. Blood. (2017) 129:1095–102. doi: 10.1182/blood-2016-09-692541, PMID: 28115369

[2] Chiba S Sakata-Yanagimoto M. Advances in understanding of angioimmunoblastic T-cell lymphoma. Leukemia. (2020) 34:2592–606. doi: 10.1038/s41375-020-0990-y, PMID: 32704161 PMC7376827

[3] Federico M Rudiger T Bellei M Nathwani BN Luminari S Coiffier B. Clinicopathologic characteristics of angioimmunoblastic T-cell lymphoma: analysis of the international peripheral T-cell lymphoma project. J Clin Oncol Off J Am Soc Clin Oncol. (2013) 31:240–6. doi: 10.1200/JCO.2011.37.3647, PMID: 22869878 PMC3532394

[4] Advani RH Skrypets T Civallero M Spinner MA Manni M Kim WS. Outcomes and prognostic factors in angioimmunoblastic T-cell lymphoma: final report from the international T-cell project. Blood. (2021) 138:213–20. doi: 10.1182/blood.2020010387, PMID: 34292324 PMC8493974

[5] International Non-Hodgkin's Lymphoma Prognostic Factors Project. A predictive model for aggressive non-Hodgkin's lymphoma. N Engl J Med. (1993) 329:987–94. 8141877 10.1056/NEJM199309303291402

[6] Gallamini A Stelitano C Calvi R Bellei M Mattei D Vitolo U. Peripheral T-cell lymphoma unspecified (PTCL-U): a new prognostic model from a retrospective multicentric clinical study. Blood. (2004) 103:2474–9. doi: 10.1182/blood-2003-09-3080, PMID: 14645001

[7] Tokunaga T Shimada K Yamamoto K Chihara D Ichihashi T Oshima R. Retrospective analysis of prognostic factors for angioimmunoblastic T-cell lymphoma: a multicenter cooperative study in Japan. Blood. (2012) 119:2837–43. doi: 10.1182/blood-2011-08-374371, PMID: 22308294

[8] Okadome K Baba Y Yagi T Kiyozumi Y Ishimoto T Iwatsuki M. Prognostic nutritional index, tumor-infiltrating lymphocytes, and prognosis in patients with esophageal Cancer. Ann Surg. (2020) 271:693–700. doi: 10.1097/SLA.0000000000002985, PMID: 30308614

[9] Zhang L Ma W Qiu Z Kuang T Wang K Hu B. Prognostic nutritional index as a prognostic biomarker for gastrointestinal cancer patients treated with immune checkpoint inhibitors. Front Immunol. (2023) 14:1219929. doi: 10.3389/fimmu.2023.1219929, PMID: 37545502 PMC10401046

[10] Chen N Yu Y Shen W Xu X Fan Y. Nutritional status as prognostic factor of advanced oesophageal cancer patients treated with immune checkpoint inhibitors. Clin Nutr. (2024) 43:142–53. doi: 10.1016/j.clnu.2023.11.030, PMID: 38043419

[11] Mourad N Mounier N Brière J Raffoux E Delmer A Feller A. Clinical, biologic, and pathologic features in 157 patients with angioimmunoblastic T-cell lymphoma treated within the Groupe d'Etude des Lymphomes de l'Adulte (GELA) trials. Blood. (2008) 111:4463–70. doi: 10.1182/blood-2007-08-105759, PMID: 18292286 PMC2343588

[12] Onodera T Goseki N Kosaki G. Prognostic nutritional index in gastrointestinal surgery of malnourished cancer patients. Nihon Geka Gakkai Zasshi. (1984) 85:1001–5. 6438478

[13] Ma S Zhang B Lu T Li D Li T Shen Z. Value of the prognostic nutritional index (PNI) in patients with newly diagnosed, CD5-positive diffuse large B-cell lymphoma: a multicenter retrospective study of the Huaihai lymphoma working group. Cancer. (2022) 128:3487–94. doi: 10.1002/cncr.34405, PMID: 35932292

[14] Wang HB Xu XT Tian MX Ding CC Tang J Qian Y. Prognostic values of the prognostic nutritional index, geriatric nutritional risk index, and systemic inflammatory indexes in patients with stage IIB-III cervical cancer receiving radiotherapy. Front Nutr. (2023) 10:1000326. doi: 10.3389/fnut.2023.1000326, PMID: 36937347 PMC10017984

[15] Wei C Li W Qin L Liu S Xue C Ren K. Clinicopathologic characteristics, outcomes, and prognostic factors of angioimmunoblastic T-cell lymphoma in China. Cancer Med. (2023) 12:3987–98. doi: 10.1002/cam4.5248, PMID: 36106610 PMC9972121

[16] de Leval L Parrens M Le Bras F Jais JP Fataccioli V Martin A. Angioimmunoblastic T-cell lymphoma is the most common T-cell lymphoma in two distinct French information data sets. Haematologica. (2015) 100:e361–4. doi: 10.3324/haematol.2015.126300, PMID: 26045291 PMC4800690

[17] Zitvogel L Pietrocola F Kroemer G. Nutrition, inflammation and cancer. Nat Immunol. (2017) 18:843–50. doi: 10.1038/ni.3754, PMID: 28722707

[18] Yao N Hou Q Zhang S Xiao H Liang Y Xu X. Prognostic nutritional index, another prognostic factor for extranodal natural killer/T cell lymphoma, nasal type. Front Oncol. (2020) 10:877. doi: 10.3389/fonc.2020.00877, PMID: 32637354 PMC7317673

[19] Luan C Wang F Wei N Chen B. Prognostic nutritional index and the prognosis of diffuse large b-cell lymphoma: a meta-analysis. Cancer Cell Int. (2020) 20:455. doi: 10.1186/s12935-020-01535-x, PMID: 32973400 PMC7493866

[20] Ge J Lei Y Wen Q Zhang Y Kong X Wang W. The prognostic nutritional index, an independent predictor of overall survival for newly diagnosed follicular lymphoma in China. Front Nutr. (2022) 9:981338. doi: 10.3389/fnut.2022.981338, PMID: 36276809 PMC9579693

[21] Periša V Zibar L Knezović A Periša I Sinčić-Petričević J Aurer I. Prognostic nutritional index as a predictor of prognosis in patients with diffuse large B cell lymphoma. Wien Klin Wochenschr. (2017) 129:411–9. doi: 10.1007/s00508-016-1077-7, PMID: 27637206

[22] Gleeson M Peckitt C To YM Edwards L Oates J Wotherspoon A. CHOP versus GEM-P in previously untreated patients with peripheral T-cell lymphoma (CHEMO-T): a phase 2, multicentre, randomised, open-label trial. Lancet Haematol. (2018) 5:e190–200. doi: 10.1016/S2352-3026(18)30039-5, PMID: 29703335 PMC5946805

[23] Horwitz S O'Connor OA Pro B Illidge T Fanale M Advani R. Brentuximab vedotin with chemotherapy for CD30-positive peripheral T-cell lymphoma (ECHELON-2): a global, double-blind, randomised, phase 3 trial. Lancet. (2019) 393:229–40. doi: 10.1016/S0140-6736(18)32984-2, PMID: 30522922 PMC6436818

[24] Moskowitz AJ Ghione P Jacobsen E Ruan J Schatz JH Noor S. A phase 2 biomarker-driven study of ruxolitinib demonstrates effectiveness of JAK/STAT targeting in T-cell lymphomas. Blood. (2021) 138:2828–37. doi: 10.1182/blood.2021013379, PMID: 34653242 PMC8718625

