# Supplementary material for: Low prognostic nutritional index predicts poor outcome in newly diagnosed angioimmunoblastic T-cell lymphoma
Source: Front Nutr. 2025 Jun 26;12:1622691. doi: 10.3389/fnut.2025.1622691 (PMC12240789; doi:10.3389/fnut.2025.1622691)

**Supplemental information**

Figure S1. The optimal cutoff value for the prognostic nutritional index using maximally selected rank statistics analysis.

grps indicates groups.


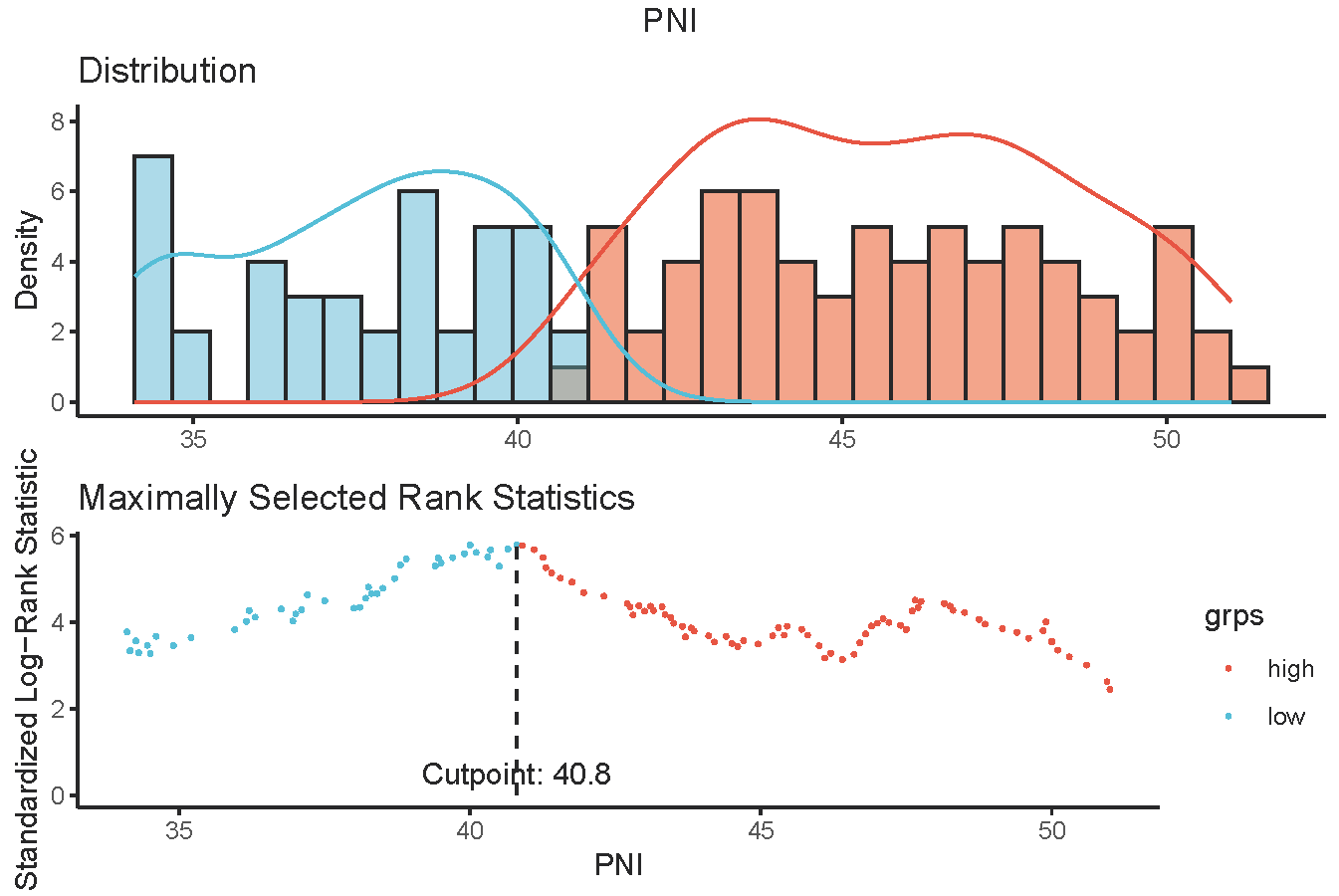


Figure S2 Forest plots of univariate and multivariate Cox regression analysis. The PNI is as an independently prognostic factor for OS and PFS.


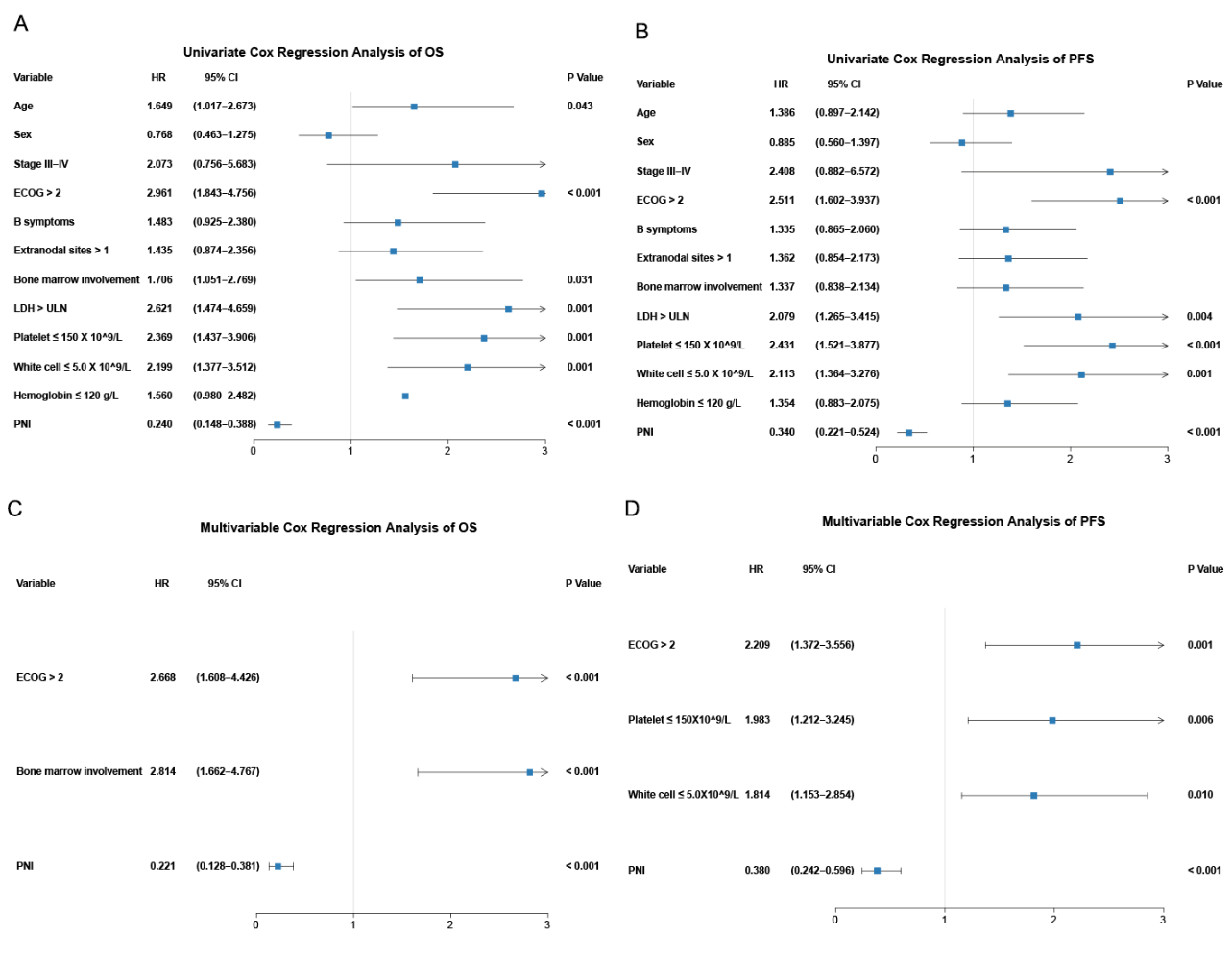

Supplement: Supplementary file 1 [file Table_1.docx]
